# Supercritical CO_2_ Synthesis of Porous Metalloporphyrin Frameworks: Application in Photodynamic Therapy

**DOI:** 10.1021/acs.chemmater.2c03018

**Published:** 2023-01-30

**Authors:** Márta Kubovics, Oriol Careta, Oriol Vallcorba, Guillermo Romo-Islas, Laura Rodríguez, Jose A. Ayllón, Concepción Domingo, Carme Nogués, Ana M. López-Periago

**Affiliations:** † Institute of Materials Science of Barcelona (ICMAB-CSIC), Campus UAB s/n, 08193Bellaterra, Spain; ‡ Department de Biologia Cel·lular, Fisiologia i Immunologia. Universtitat Autònoma de Barcelona (UAB), Campus UAB s/n, 08193Bellaterra, Spain; § ALBA Synchrotron Light Source, 08290Cerdanyola del Vallés, Spain; ∥ Department of Inorganic and Organic Chemistry, Barcelona University, Martí i Franquès 1-11, 08028Barcelona, Spain; ⊥ Institute of Nanoscience and Nanotechnology (IN2UB), Barcelona University, Campus UB s/n, 08028Barcelona, Spain; # Department de Química, Universtitat Autònoma de Barcelona (UAB), Campus UAB s/n, 08193Bellaterra, Spain

## Abstract

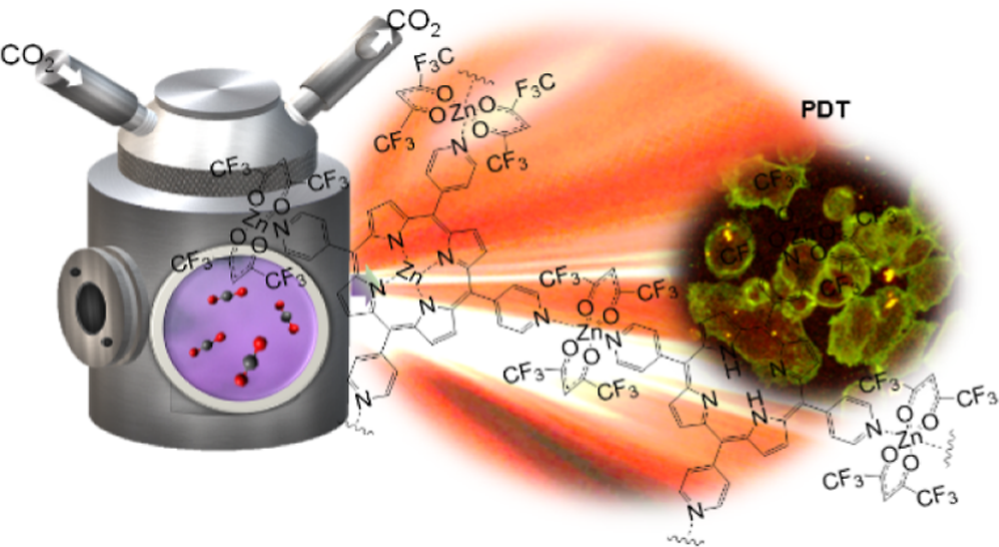

A series of porous metalloporphyrin frameworks prepared from the 5,10,15,20-tetra(4-pyridyl)porphyrin (H_2_TPyP) linker and four metal complexes, M(hfac)_2_ M = Cu(II), Zn(II), Co(II), and Ni(II) (hfac: 1,1,1,5,5,5-hexafluoroacetylacetonate), were obtained using supercritical CO_2_ (scCO_2_) as a solvent. All the materials, named generically as [M-TPyP]_*n*_, formed porous metal–organic frameworks (MOFs), with surface areas of ∼450 m^2^ g^–1^. All MOFs were formed through the coordination of the metal to the exocyclic pyridine moieties in the porphyrin linker. For Cu(II), Zn(II), and Co(II), incomplete metal coordination of the inner pyrrole ring throughout the structure was observed, giving place to MOFs with substitutional defects and leading to a certain level of disorder and limited crystallinity. These samples, prepared using scCO_2_, were precipitated as nano- to micrometric powders. Separately, a layering technique from a mixture of organic solvents was used to crystallize high-quality crystals of the Co(II) based MOF, obtained with the formula [{Co(hfac)_2_}_2_H_2_TPyP]_*n*_. The crystal structure of this MOF was elucidated by single-crystal synchrotron X-ray diffraction. The Zn(II)-based MOF was selected as a potential photodynamic therapy drug in the SKBR-3 tumoral cell line showing outstanding performance. This MOF resulted to be nontoxic, but after 15 min of irradiation at 630 nm, using either 1 or 5 μM concentration of the product, almost 70% of tumor cells died after 72 h.

## 1 Introduction

Porphyrins are key building block precursors in many areas of synthetic chemistry. As an important example, the preparation of metal–organic frameworks (MOFs) containing porphyrin linkers is currently of great interest as they can form coordination networks with multiple applications such as catalysis, sensors for molecular recognition,^1−3^ or drugs for cancer treatment.^4−6^ In the latter, porphyrins are the most extensively used material for photodynamic therapy (PDT).^7^ The PDT procedure is based on the local application of a photosensitizer in the affected area that, by light radiation of a certain wavelength, induces the formation of reactive oxygen species (ROS)^8^ able to destroy the harmful cells through either necrosis or apoptosis.^9^ In this technique, the use of net porphyrins is often limited by their low biostability in aqueous media, self-aggregation, and nonselective tumor targeting.^10^ Some of these drawbacks can be significantly attenuated by building porous structures in which the porphyrins are the organic linkers located between metal-containing species, thus obtaining porphyrin-based MOFs. From them, the so-called fourth generation of photosensitizers is built,^11^ where the porphyrin moieties are somehow isolated and their aggregation is hindered.^12^ The first report on PDT using porphyrins as building blocks in MOFs involves the use of Hf(IV)^13^ and Zr(IV)^14^ as metal centers for head and neck cancer and cervical cancer, respectively.

Among the most studied porphyrin MOFs are those involving Zr(IV), giving high stability and structural diversity,^15^ Ti(IV), applied in photocatalysis,^16^ and divalent Zn(II), Co(II), and Cu(II) cations, forming usually very stable 2D sheet-like structures.^17−19^ Less common, but equally functional, are the porphyrin-based MOFs with pyridyl motifs that can form supramolecular structures through self-assembly.^20^ In particular, 5,10,15,20-tetra(4-pyridyl)porphyrin (H_2_TPyP), the linker chosen in this study, possesses several potential coordination sites. The inner tetratopic unit can be coordinated by displacing the two protons of the N–H pyrrole subunits, and the exocyclic four pyridyl groups linked to the porphyrin ring can potentially coordinate from one to four metal ions, which can greatly enrich the structure and functionality. Nevertheless, not many examples of MOF constructions are found in the literature for this type of pyridyl-based porphyrin linkers, presumably because H_2_TPyP has an extremely low solubility in most conventional liquid solvents, which is difficult enormously for their processing. In fact, H_2_TPyP is only partially soluble in chloroform, dimethylformamide, and trichloroethane. Examples of total metal complexation are the coordination of Zn(II) ions with the pyridyl groups of adjacent H_2_TPyP molecules and the same structure but with Ag(II) coordinated to the pyrrole units forming 2D coordination polymers.^21^ Two other examples of full coordination in TPyP^2–^ were obtained using Cu(II) and consisted of porous 2D coordination networks involving the paddlewheel Cu(II) tetraacetate [Cu_2_(AcO)_4_] secondary building unit^22^ or the Cu(hfac)_2._^23^ In both cases, Cu(II) fulfilled different tasks since it acts as a metal source to metalize the porphyrin ring by interchanging Cu(II) with the two central protons of the NH pyrrole ring and as a neutral node maintaining the ligands AcO or hfac.

The described H_2_TPyPs-based MOFs have been obtained using long-lasting synthetic methods (from weeks to months), either using layering (solvent diffusion) or solvothermal approaches. These techniques produce high-quality crystals proper for single-crystal X-ray diffraction (XRD) resolution but in extremely small yields. Besides, these approaches can have the limitation of solvent incorporation into the pores of the MOF, which can modify the structure of the final product. Currently, the synthesis of MOFs is beyond the study of their fundamental framework as in many circumstances, developing key applications does not necessarily imply having well-resolved crystal structures. In fact, amorphous or semiamorphous MOFs have also shown a high capability to perform certain applications, such as in drug delivery systems.^24^ In this way, the search for new preparation methods involving short reaction times, low temperatures, and avoiding toxic solvents, while achieving large yields, is under strong surveillance, even if the quality of the crystals is partially compromised. Among the different techniques developed to synthesize MOFs and coordination polymers, those based on supercritical CO_2_ (scCO_2_) have proven to be excellent for precipitating materials in high yields. scCO_2_ has been traditionally used for removing entrapped solvents within MOF structures. However, in the past few years, it has also been used in our research group as a solvent for the synthesis of MOFs, either by using exclusively scCO_2_,^25−27^ or adding a cosolvent,^28^ with interesting applications in medicine (bioMOFs),^29^ sensors^30^ or gas adsorption after hybridizing with graphene aerogels.^31^ In essence, the use of the scCO_2_ technology has proved excellent to prepare materials for biomedical applications.^32^ In the search for new applications, and as a part of ongoing work, we have explored here the possibilities of precipitating MOFs based on the H_2_TPyP porphyrin linker in scCO_2_. Four metal hexafluoroacetylacetonate complexes [M(hfac)_2_, M = Cu(II), Zn(II), Co(II), and Ni(II)] were used to complete the MOF structure, using only scCO_2_ as a solvent or adding a small amount of chloroform as a cosolvent, when necessary. In order to obtain crystals for the structural elucidation, a layering approach was attempted for all the studied products. However, this approach only allowed attaining proper crystals for the MOF involving Co(II), which was further elucidated in regard to the crystalline structure. Finally, the scCO_2_ precipitated Zn(II) MOF was successfully tested as a potential photosensitizer in PDT.

## 2 Materials and Methods

### 2.1 Materials

The organic linker 5,10,15,20-tetra(4-pyridyl)porphyrin; the metallic units of copper, zinc, cobalt, and nickel hexafluoroacetylacetonate ([Cu(hfac)_2_]·*x*H_2_O, [Zn(hfac)_2_]·2H_2_O, [Co(hfac)_2_]·*x*H_2_O, and [Ni(hfac)_2_]·*x*H_2_O); the solvents chloroform (CHCl_3_), methanol (MeOH), and 1,1,2-trichloroethane (Cl_3_Et); and sodium anthracene-9,10-dipropionic acid (Na-ADPA) and perinaphthenone, used for the singlet oxygen measurement, were all purchased from Sigma-Aldrich. CO_2_ (99.995%) was supplied by Carburos Metálicos S.A., Air Products Group (Spain).

### 2.2 Methods

#### 2.2.1 Synthesis of MOFs

##### 2.2.1.1 scCO_2_ Synthesis

[Zn-TPyP]_*n*_, [Cu-TPyP]_*n*_, and [Ni-TPyP]_*n*_ MOFs were obtained in pure scCO_2._ Reactions were performed in a 10 mL vial placed into a 100 mL high-pressure autoclave with two opposite sapphire windows (Thar Design). The vial was charged with 0.34 mol of the metal complex M(hfac)_2_ and 0.11 mol of the organic linker, standardizing a molar ratio of ca. 3.1:1 for M(II)/H_2_TPyP in all cases. A small magnetic bar was also added, and the vial was finally capped with cellulose filter paper. In case of [Co-TPyP]_*n*_, ∼5 v % of CHCl_3_ was also added as a cosolvent. Experiments were performed using scCO_2_ at 20 MPa and 60 °C, stirring the powder into the vial at 500 rpm, and for 72 h. After this running time, samples were cleaned with a flow of fresh CO_2_ at high pressure to remove the excess of unreacted M(hfac)_2_. Finally, the reactor was slowly depressurized and cooled down to room temperature, to recover in all cases a fine garnet-colored dry powder, in an average yield of 85 wt %.

##### 2.2.1.2 Layering

Crystallization assays using the layering approach were tried for all studied metals. However, only the one involving Co(II) successfully produced crystals available for structural elucidation. For this experiment, H_2_TPyP (0.025 mmol) was dissolved in 10 mL of Cl_3_Et/MeOH (3:1) and layered with 2 mL of fresh MeOH. A solution of [Co(hfac)_2_(H_2_O)_*x*_] (0.104 mmol) in 10 mL of MeOH was carefully layered onto the pristine MeOH layer. After several weeks at room temperature, purple needle-like crystals precipitated. The crystals were carefully filtered, rinsed with fresh MeOH, and finally dried under vacuum.

### 2.3 Characterization

The structure of the obtained samples was analyzed by powder XRD (PXRD) using a Siemens D5000 X-ray powder diffractometer using the Cu Kα incident radiation. The diffraction patterns were recorded from 2θ = 5 to 30°, with a step scan of 0.02° counting for 1 s at each step. The morphological analysis was performed using a Quanta FEI 200 FEG-ESEM scanning electron microscope. The thermal properties of the samples were evaluated by thermogravimetric analysis (TGA, PerkinElmer 7). Measurements were carried out under a N_2_ atmosphere, raising the temperature at a rate of 10 °C min^–1^. Fourier transform infrared (FTIR) spectra of the solid samples dispersed in KBr were recorded on a PerkinElmer Spectrum apparatus. The textural properties were determined by N_2_ adsorption/desorption experiments, performed at −196 °C, using ASAP 2020 Micromeritics Inc equipment. The samples were previously degassed at 120 °C for 24 h. The Brunauer–Emmet–Teller (BET) surface area (*S*_BET_) of the precipitated compounds was estimated in the relative pressure range of ca. 0.05–0.20. The micropore Langmuir surface (*S*_Langmuir_) was also calculated, together with the micropore volume (*V*_mp_) estimated by the t-method. Elemental analysis was performed using a Flash EA2000 Thermo Fisher Scientific analyzer. Ultraviolet–visible (UV–vis) absorption measurements in water were carried out using a Varian Cary 5000 apparatus with an operational range in the spectrophotometer of 190–3300 nm. Dynamic light scattering (Zetasizer Nano ZS Malvern Inst.) was used to measure the hydrodynamic size.

Single-crystal XRD (SCXRD) experiments for [{Co(hfac)_2_}_2_TPyP]_*n*_ were performed in the XALOC beamline at the ALBA synchrotron (Spain). Data were collected at 100 K with a 0.72931 Å wavelength using the Dectris Pilatus 6M detector placed at 120 mm from the sample. Nine scans were performed from 0 to 360° in steps of 0.5° with a collection time of 0.15 step^–1^. The scan was repeated at three different N angles (0, 45, and 90°) and merged afterward to increase the completeness and redundancy when possible. Data were indexed, integrated, and scaled using the XDS software.^33^ The crystal structures were solved by intrinsic phasing and refined with SHELXL (version 2014/7)^34^ using Olex2 as a graphical interface.^35^

### 2.4 Measurement of Singlet Oxygen (^1^O_2_) Production

#### 2.4.1 Singlet Oxygen Detection via Fluorescence Decay of ADPA

Singlet oxygen formation was detected using a Cary Eclipse spectrofluorometer (Agilent). The process involved measuring the fluorescence decay of ADPA upon irradiation in the presence of the [Zn-TPyP]_*n*_ photosensitizer.^36^ For this, 100 μL of a Na-ADPA water solution (1.2 mM) was added to 3 mL of [Zn-TPyP]_*n*_ dispersion in water (0.15 mg mL^–1^). The mixture was irradiated at 630 nm for 15 min using the same light source as for the photodynamic treatment (PhotoActivation Universal Light PAUL device, GenIUL). After each 5 min, the mixture was introduced to a quartz cuvette of 1 cm, and the fluorescence emission spectra of the mixture were recorded in the 380–600 nm range, using an excitation wavelength of 355 nm.

#### 2.4.2 Singlet Oxygen Quantum Yield (ϕ_Δ_) Measurement

Room-temperature singlet oxygen phosphorescence was detected at 1270 nm with a Horiba-Jobin-Ybon SPEX Nanolog spectrofluorometer using a DSS-IGA020L detector. The use of a Schott RG 1000 filter was essential to eliminate from the infrared signal all the first harmonic contribution of sensitizer emission in the region below 850 nm. The singlet oxygen formation quantum yield was then determined by direct measurement of the phosphorescence at 1270 nm following irradiation of the aerated aqueous dispersions of the samples. Perinaphthenone in dichloromethane was used as a standard reference, applying eq 1.
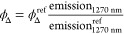
1where _Δ_^ref^ is the singlet oxygen formation quantum yield of the reference compound (.

### 2.5 Cell Culture Experiments of [Zn-TPyP]_*n*_

Biological experiments were performed using the SKBR-3 cell line, a tumorigenic human mammary epithelial cell line (ATCC, USA). Cell culture was carried out in McCoy’s 5A modified medium (Gibco) supplemented with 10% fetal bovine serum (Gibco) in a 37 °C humidified incubator set to 5% CO_2_ (standard culture conditions). For both analysis and toxicity evaluation in the dark and under irradiation (photodynamic experiments), cells were seeded in 4- or 24-well plates at a density of 3.5 × 10^4^ cells per well. For confocal laser scanning microscopy (CLSM) experiments, cells were seeded in special confocal 35 mm dishes provided with a glass coverslip bottom (μ-Dish 35 mm, high Glass Bottom, Ibidi GmbH) at a density of 1.75 × 10^5^ cells per well. All product treatments were performed 24 h after cell seeding. Stock product dispersions were prepared in filtered distilled water to a final concentration of 1 mM. Then, stock solutions were diluted in cell media, obtaining a final product concentration of 1, 5, and 10 μM.

#### 2.5.1 Dark Toxicity Evaluation

The toxicity of the product in the absence of irradiation (from now on, dark toxicity) was evaluated by determining cell viability at two different time points (24 and 72 h postincubation) by the Alamar Blue assay. In brief, 24 h after cell seeding, cells were incubated for 24 h with different concentrations of product [0 (control), 1, 5, and 10 μM] previously treated with an ultrasonic bath. Later, the cell medium was removed, and cells were washed four times with Hank’s balanced salt solution (HBSS, BioWest) to remove any product remains, followed by fresh medium addition. Viability was assessed twice, right after medium addition (viability at 24 h) or after an additional 48 h incubation in standard culture conditions (viability at 72 h). Three independent experiments were performed for each condition.

#### 2.5.2 Photodynamic Treatments

Photodynamic treatments were performed following the same protocol as for the dark toxicity, but this time, SKBR-3 cells were incubated with 1 or 5 μM products for 4 h. After 4 h of incubation, the cell medium was removed, and cells were washed four times with HBSS to remove any product remains, followed by fresh medium addition. Later, cells were either kept in dark conditions (not irradiated) or irradiated for 15 min using a PhotoActivation Universal Light device (PAUL, GenIUL) in the range of 620–630 nm (red light) and with a mean intensity of 55 mW cm^–2^ (light dose of 33 J cm^–2^). Cell culture viability assessments were performed twice, right after irradiation (viability at 24 h) or after being incubated for an additional 48 h in standard conditions (viability at 72 h), using the Alamar Blue assay. Three independent experiments were performed for each condition.

#### 2.5.3 Alamar Blue Assay

Cell viability was determined using the Alamar Blue cell viability reagent (Thermo Fisher Scientific). In brief, after product incubation either during 4 or 24 h, the cell medium was removed, and cells were washed four times with HBSS before adding 10% Alamar Blue to the fresh medium. Cells were then incubated for 4 h in standard conditions in the dark. After incubation, the medium was collected, and 200 μL of the solution was transferred to a black-bottom Greiner CELLSTAR 96-well plate (Sigma-Aldrich). Finally, the fluorescence of the medium was measured at a 590 nm wavelength after excitation at 560 nm on a Spark multimode microplate reader (Tecan). Three independent experiments were performed for each condition.

#### 2.5.4 Product Internalization

To visualize if there was any product internalization, 1.75 × 10^5^ cells were seeded on confocal 35 mm dishes provided with a glass coverslip bottom. After 24 h of cell seeding, 1 μM product was added to the culture. After product incubation (4 or 24 h) in standard conditions in the dark, cells were washed four times with HBSS to remove the noninternalized product and incubated with 5 μL of wheat germ agglutinin (WGA) 448 for 15 min to detect the limit of the cells. Then, images were acquired using a Leica TCS-SP5 AOBS spectral confocal laser scanning microscope (Leica Microsystems) using a PlanApochromatic 63× objective lens. WGA-Alexa Fluor 488 (plasma membrane) and product excitation was carried out using 405 and 488 nm laser lines, respectively, using a sequential mode. Different PMT devices [600–763 nm for product fluorescence emission and 500–550 nm for WGA fluorescence emission (membrane)] were used to detect each corresponding spectral range. A series of images were further analyzed with Fiji software (ImageJ–NIH).

#### 2.5.5 Statistical Analyses

The statistical analysis was performed using GraphPad Prism version 6.01 for Windows (GraphPad Software). Quantitative results were analyzed using a two-way ANOVA with a minimal significance level set at *p* ≤ 0.05. In the figures, significance is represented by an asterisk, which means that the values are significantly different from their control (*p* < 0.05).

## 3 Results and Discussion

### 3.1 scCO_2_ Synthesis

It is generally recognized that when preparing MOFs, the solvent of choice is highly important since it can affect several parameters, such as structure, morphology, crystallinity, and particle size of the end product. As mentioned in the Introduction section, reported MOFs involving H_2_TPyP as a linker have been obtained using techniques where the yield was compromised toward obtaining high-quality crystals. This work explores the use of green scCO_2_ as an efficient solvent for the preparation of new H_2_TPyP-based MOFs at a high yield and in the absence of large amounts of toxic solvents. For achieving this target, hexafluoacetylacetonate metal ligands (M(hfac)_2_), with typical high solubility in scCO_2_, were used. In this method, the use of neutral metallic nodes is preferred due to the limited capacity of scCO_2_ for dissolving ionic materials. With scCO_2_ being the solvent used in this work for product synthesis, reagent solubility in this fluid is an important parameter determining product characteristics. Reagent solubility values in this fluid were first empirically estimated by a simple visual method carried out in the sapphire windows reactor. Weighed amounts of micronized powder were added to the reactor that was then filled with scCO_2_ under typical reaction conditions chosen for MOF synthesis (e.g., 20 MPa, 60 °C). The turbidity of the system, maintained under vigorous stirring, was observed for periods of several hours. Only for the H_2_TPyP linker, the turbidity was permanent, indicating very little solubility in scCO_2_. In regard to the metal precursors, experimentally, it was observed that solubility decreases as follows: Co(hfac)_2_ > Zn(hfac)_2_ ∼ Cu(hfac)_2_ > Ni(hfac)_2_. The solubility values of these substances have been compiled in the literature,^37^ but they are not totally coincident with our observations, likely due to the different levels of hydration of the reagents. Tests performed in this work pointed out Ni(hfac)_2_ having the lowest solubility value compared to the other metal precursors, concurrent with its high hydration level.

Since some of the used reagents were not highly soluble in scCO_2_, the process was first optimized by adjusting the synthetic parameters to avoid the presence of large amounts of unreacted molecules after MOF precipitation, which can contaminate the end product. The first trial consisted in using a large excess of M(hfac)_2_ with respect to the organic linker (molar ratio 6:1). However, no significant changes in regard to the structure or purity of the end product were observed when comparing this ratio to the stoichiometric molar ratio corresponding to the addition of enough metal for reaching full complexation of all the potential binding sites in the H_2_TPyP, for example, the four pyridine groups and the inner pyrrole ring. Accordingly, further trials were carried out using a 3.1:1 M(hfac)_2_/porphyrin molar ratio, aiming to also minimize the presence of remaining M(hfac)_2_ after MOF precipitation. An exception to this rule was the precipitation of the MOF of Co(II), for which whatever the experimental conditions, large amounts of unreacted H_2_TPyP were always present in the end product. Thus, to precipitate this MOF, the CH_3_Cl cosolvent was employed to enhance linker solubility. Initial experiments were carried out lasting 3 h, but after this short period of time, mixtures of end products and unreacted H_2_TPyP were always recovered. Hence, the influence or reaction time was studied by increasing the reaction time in steps, up to a week. The withdrawal of the diffraction peaks of the pure porphyrin linker in the PXRD patterns was chosen as the standard indication of the evolution of the H_2_TPyP toward the end product. Upon analyzing the results, after 72 h, no further changes in the pattern were observed; thus, the reaction time was standardized at this time for all of the metals to complete the reaction.

### 3.2 Structure of the Synthetized MOFs

Several attempts were performed to synthesize high-quality crystals of all the studied porphyrin-derived MOFs. Used techniques were layering and the solvothermal approach, in which the reagents were kept at 120 °C in dimethylformamide in closed vials for 72 h. From all of the performed trials, only the layering of Co(II) and H_2_TPyP in Cl_3_Et/MeOH resulted in crystals of the necessary quality for performing structural elucidation by SCXRD. From the rest, only fine particles were precipitated, characterized by PXRD (Figure S1), and not corresponding to the phases precipitated in scCO_2_. The characteristics of the rest of the scCO_2_-precipitated new compounds were determined by comparison with the PXRD pattern of the Co(II)-derived porphyrin, either prepared by the scCO_2_ method or simulated from the single-crystal structural data.

#### 3.2.1 [{Co(hfac)_2_}_2_H_2_TPyP]_*n*_ Structure

**Figure 1:**
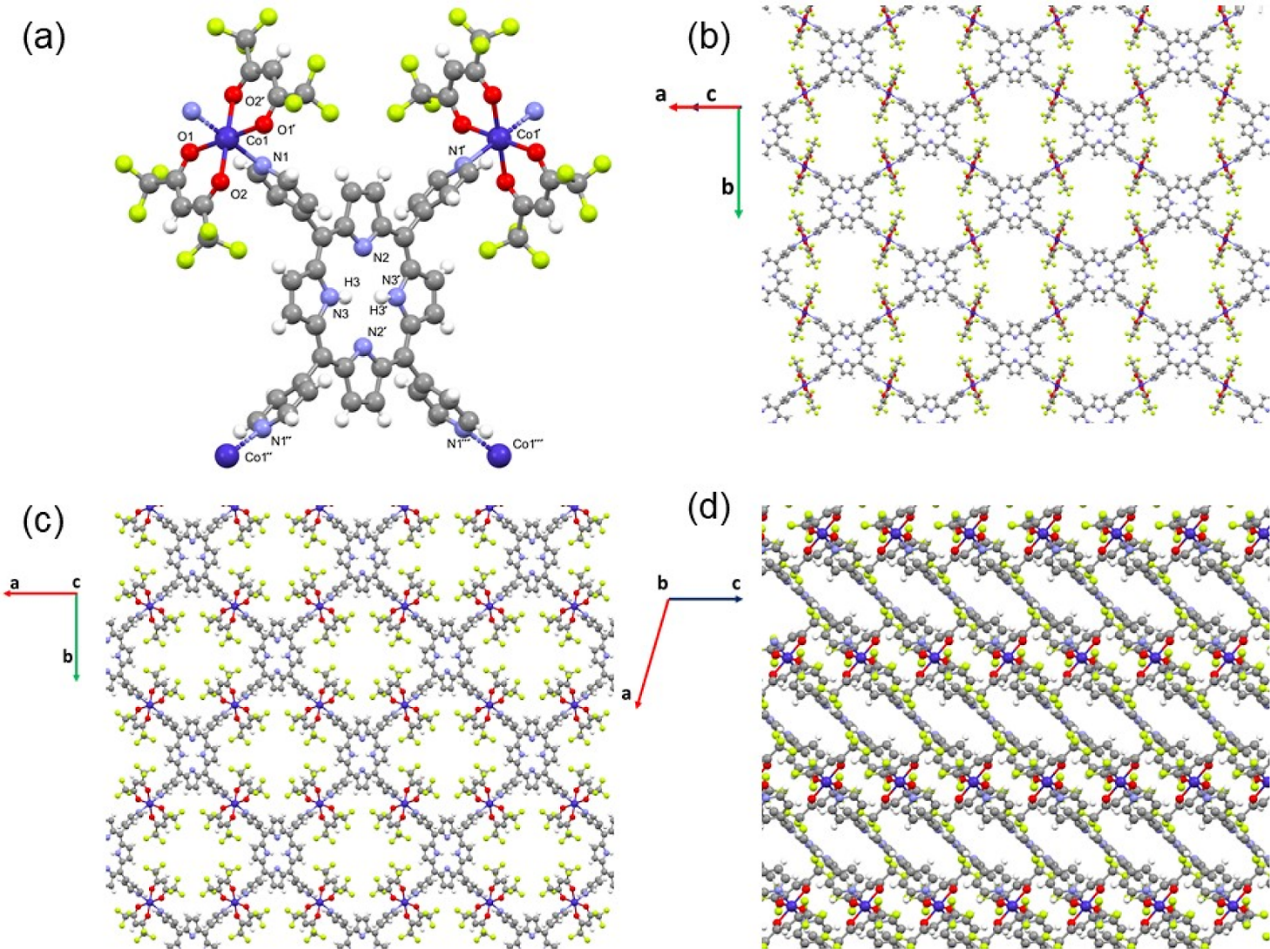
Plots of the crystal structure of compound [{Co(hfac)_2_}H_2_TPyP]_*n*_: (a) numbering atoms, (b,c) showing different plane orientations, and (d) stacking.

The porphyrin-derived MOF of Co(II) precipitated from layering, with the formula [{Co(hfac)_2_}_2_H_2_TPyP]_*n*_, does not show the metal coordination in the inner porphyrin ring, as shown in Figure 1a. Crystallographic data is presented in Table S1. The four pyridines coordinated into metal clusters forming a polymeric structure. This synthesis is believed to be thermodynamically and kinetically favored. The coordination of the pyridines is a highly favorable process because the exocyclic groups are highly exposed toward the approach of the metal clusters, and the orbital geometry of the nitrogen is also favorable. In addition, Co(hfac)_2_ can coordinate the pyridine without the elimination of any of its hfac moieties, thus maintaining the ligands and reducing the necessary reaction energy. This addition process occurs in one step, without the release of any component, and therefore, it can occur in a fast and effective way. On the contrary, the use of large metallic clusters might sterically hinder the access of the Co(hfac)_2_ units to the inner pyrrole ring. Furthermore, the coordination to the pyrrolic ring would imply the substitution and elimination of the proton in the pyrrole (N–H) and the elimination of the hfac moiety as hexafluoroacetilcetone upon metal coordination. Overall, the metal coordination of the pyrrolic ring is a highly energetic process and therefore less probable to occur in comparison to the pyridine M(hfac)_2_ addition. Since the synthesis of the Co(II) MOF is performed at room temperature, it can be assumed that, in the apparent competing reactions pyridine–metal versus pyrrole–metal, the lack of temperature as energy input is detrimental for the latter versus the former.

[{Co(hfac)_2_}_2_H_2_TPyP]_*n*_ belongs to the monoclinic crystal system, with the space group *C*2/*m* (12). The asymmetric unit contains one cobalt atom, one hfac ligand, and a quarter of the porphyrin linker. The crystal structure resembles that previously described for [{Cu(hfac)_2_}CuTPyP·6H_2_O]_*n*_,^23^ in particular on the framework structure, space group, and cell parameters. However, the Co(II) compound in this work shows three key differences: (i) it contains Co(hfac)_2_ nodes instead of Cu(hfac)_2_ nodes, (ii) the porphyrin is nonmetalated, and (iii) the pores do not contain any solvent. The structural analysis revealed a 2D sheet coordination polymer comprising trans-configured Co(hfac)_2_ nodes, joined by four-connected H_2_TPyP, acting as tetratopic linkers, through the four pyridyl groups. The ensemble defines a (4,4)-net with a rhomboidal distortion from a regular square grid. The porphyrin ring system is essentially flat, although it is slightly inclined with respect to the main sheet planes defined by the Co(II) positions (angle 5.67°) (Figure 1b). The pyridyl rings are 74.07(12)° [(76.55(8)° in the copper compound] tilted relative to the mean plane of the porphyrin macrocycle. The coordination sphere about Co(II) is essentially a distorted elongated octahedron, involving four oxygen donor atoms of the chelating hfac ligands in a plane, with a Co–O bond length of 2.06 Å, and the axial positions occupied by two nitrogen atoms from pyridyl groups belonging to two different H_2_TPyP linkers, with a Co–N bond length of 2.15 Å. The bond angles about Co(II) show some deviations from the ideal 90° (values between 85 and 95°), and, besides the pyridyl ring plane, are not perpendicular to the Co–O_4_ plane 72.27° (Figure 1c). The polymeric sheets are stacked but with some offset. Therefore, the distance between the main planes of adjacent sheets, 4.84 Å, is shorter than the closer Co–Co separation, 6.52 Å (Figure 1d).

**Figure 2:**
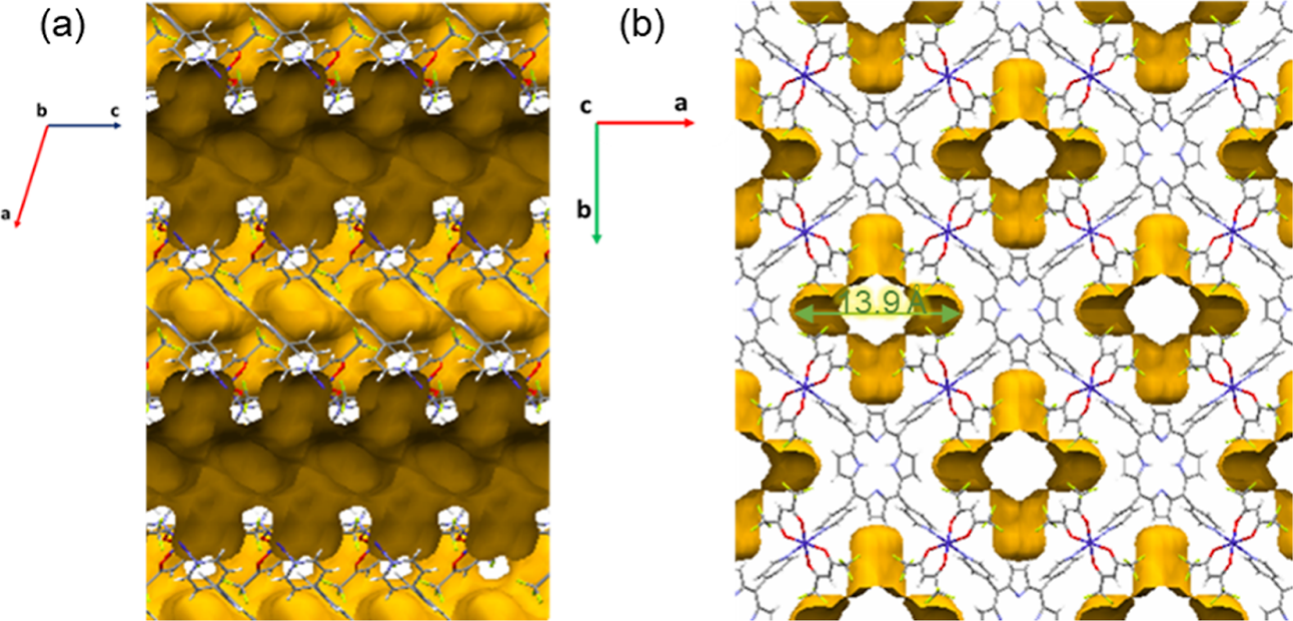
Plots of the crystal structure of compound [{Co(hfac)_2_}H_2_TPyP]_*n*_ showing the channel voids: (a) *a* and (b) *b* directions.

The stacking defines channels parallel to the *c*-axis. The channels have alternative protuberances in the *a* and *b* directions (Figure 2a,b). The estimated accessible volume is 22 v % per unit cell. Crystals are stable when subjected to humidity in air, although it is worth noting that it is very unusual for a crystal with such a high solvent-accessible volume to be stable when exposed to an open atmosphere. The pore size was measured along the *a*-axis, giving a value of 14 Å. Several weak interactions are established among layers, resulting in stable 3D crystals, for example, C–H···F contacts that involve the β-carbon pyrrole atom and CF_3_ groups of neighboring layers (H13···F2 2.542 Å), interactions between the H of the pyridine rings and O from the hfac ligand, and weak F···F contacts (Figure 1d).

#### 3.2.2 [M-TPyP]_*n*_ MOFs Prepared in scCO_2_

scCO_2_ was selected as the reaction medium for synthesizing this type of material as it plays many important roles within MOF synthesis and activation. The solubility of the reagents in different solvents guides nucleation and crystal growth kinetics, while the protic/aprotic character of the solvent determines the crystallized phase. scCO_2_ is an aprotic nonpolar solvent, with a strong quadrupole moment, which has a Lewis acid character. Moreover, it acts as an excellent solvent for neutral and fluorine-containing metal groups, which makes it an ideal solvent for M(hfac)_2_ metal clusters. Hence, the role of scCO_2_ in the synthesis of the [M-TPyP]_*n*_ MOFs is being a solvent for the metallic node added to the reaction media in the form of M(hfac)_2_. The solubility of polar compounds in scCO_2_ has historically been increased by adding small quantities of liquid cosolvents to the fluid. However, it was previously demonstrated for ZIF-8^38^ and curcumin^29^ 3D MOFs that these materials can also be precipitated in scCO_2_ being only one of the reagents, for example, the metal complex, soluble in the fluid. The formation of the [M-TPyP]_*n*_ MOFs is suggested to proceed in scCO_2_ by the interaction of dissolved M(hfac)_2_ molecules with weakly bonded H_2_-porphyrin molecules located on the surface of the porphyrin, giving place to [(M(hfac)_2_)_*x*_-H_y_TPyP] complexes and to MOF nuclei.

It is well known that the metal coordination of the pyrrolic ring provides thermodynamic stability to the structure; however, in all of the cases presented here, there was no full coordination of the pyrrolic ring. As previously explained, the formation of [M-TPyP]_*n*_ MOFs in scCO_2_ is ruled by the reactivity between the nitrogen atoms of the pyridine and the pyrrole and the metal bonded to hfac. As scCO_2_ is an aprotic solvent, the free nitrogen would not undergo protonation, and therefore, all the N (the four pyridines and the inner tetrapyrrole ring) are available for bonding in this solvent. The fact that all possible N–M bonds were not completed, even adding an excess of metal, is assumed to be related to the steric hindrance of the M(hfac)_2_ to enter the porphyrin ring and the lack of enough energy under working conditions to break the M-hfac bond. To test which moiety reacts in preference with the metal, UV–vis titration experiments in CHCl_3_ were carried out taking the Zn(II) MOF as the case study (Figure S2). For this, a solution of Zn(hfac)_2_ at different concentrations was added dropwise to a solution of H_2_TPyP. In all cases, even the most concentrated one, the four Q bands typical of the pyrrole ring remain present, thus indicating the lack of complexation that is exhibited by the declining to only two Q bands. The H_2_TPyP porphyrin has two different ways of binding to metal: (i) the metalation of the central ring, which requires simultaneously both the deprotonation of the two pyrrole groups in the porphyrin, and the substitution of the two acetylacetonate ligands in the metal, and (ii) the coordination of the pyridine groups to the metal without the need for displacing the acetylacetonate ligands. Both reactions can enter into the competition. However, it is foreseeable that the mechanism of the reaction is multistage and, in any case, more complex than the bonding of the pyridine groups necessary to form the two-dimensional network in the MOF. The obtained results in this work indicate that, in scCO_2_, the reaction produces a material with a partial degree of metalation. Hence, although both reactions compete and occur simultaneously, the bonding of the pyridine groups is favored, for example, is faster than the metalation. Probably, if a porphyrin is incorporated into the crystal lattice before being metalized in the central ring, it is highly unlikely to be metalized afterward since the metal precursor M(hfac)_2_ is too bulky to diffuse through the pores of the MOF. Another argument in favor of this hypothesis is derived from the nature of the [{Co(hfac)_2_}_2_H_2_TPyP]_*n*_ compound, obtained by the slow diffusion of reagents in organic solvents, and in which the porphyrin rings are not metalized. This indicates that, under the close-to-equilibrium experimental conditions used for obtaining single crystals, the metallic precursor is favorably consumed to form the crystal lattice bonding to the pyridine groups than to metalize the central ring of the porphyrin. It was found that, even under rapid mixing conditions, and using an excess of metallic precursor, part of the porphyrin rings has no option of incorporating metal in the central ring, and the four characteristic Q bands of the protonated pyrrole ring are always observed (Figure S2).

#### 3.2.3 Structural PXRD Analysis

**Figure 3:**
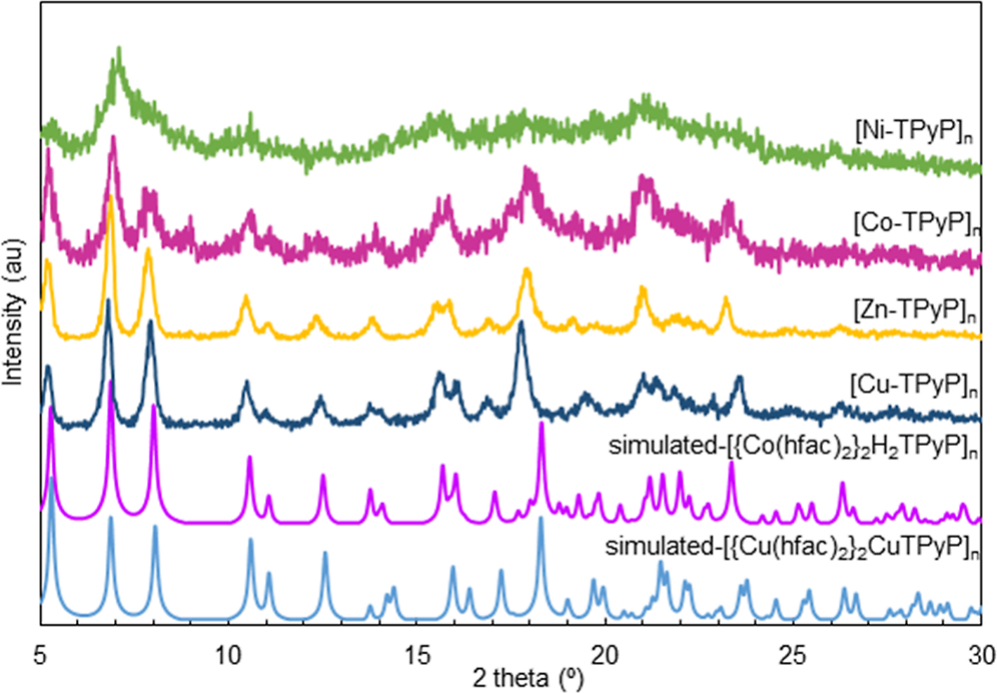
PXRD experimental patterns of [M-TPyP]_*n*_ MOFs prepared in scCO_2_ compared to the single-crystal simulated patterns of [{Cu(hfac)_2_}_2_CuTPyPx6H_2_O]_*n*_ (Nuwcak)^23^ and [{Co(hfac)_2_}_2_H_2_TPyP]_*n*_.

The PXRD patterns of the different samples are shown in Figure 3 in the 2θ range of 5 to 30°. For comparison, they are contrasted to the simulated PXRD pattern of [Co(hfac)_2_}_2_H_2_TPyP]_*n*_, resolved in this work, and to the published structure of [{Cu(hfac)_2_}_2_CuTPyP]_*n*_ (Nuwcak)^23^ that contained six molecules of water within the structure. Both, simulated PXRD patterns show three characteristic peaks at 2θ 5.2, 6.8, and 8.0°. These peaks are also present in the scCO_2_-prepared MOFs of Cu(II) and Zn(II), with a slight shift to lower angles attributed to the heterogeneity produced by the coordinated/uncoordinated pyrrole ring. Besides, most of the 2θ peaks between 10 and 18° found for the Co(II) resolved MOF and Cu(II) Nuwcaḱs were also present in the different porphyrin samples. In short, for the scCO_2_-synthesized Cu(II) and Zn(II) MOFs, PXRD analysis indicates similar diffraction peaks, as well as similar relative intensities, which is an indication of predominant isostructural features. By contrast, the scCO_2_-prepared Co(II) and Ni(II) MOFs can be considered as semiamorphous compounds when analyzing their PXRD patterns. This is a clear example of a MOF, the porphyrin-derived MOF of Co(II), which could be prepared as a fully crystalline and semiamorphous material. The possibility of obtaining pairs of semiamorphous/fully-crystallized MOFs was first described for mesoporous carboxylates of Fe(III) involving the MIL-100(Fe) and Fe-BTC pair.^24^ The semiamorphous product maintains the crystallographic structure of the crystalline counterpart, as well as a significant percentage of the porosity. It should be remarked that, for some applications, the semiamorphous material surpasses the characteristics of the crystalline product.^39^ In the scCO_2_ synthesis, semiamorphous MOFs were obtained when using the most [Co(II)] and less [Ni(II)] soluble metal reagents.

The published structure of the Nuwcak Cu(II) MOF showed a fully metal-coordinated (outer and inner ring) product, whereas the Co(II) MOF precipitated in this work does not contain any metal ion in the inner pyrrole ring. Nevertheless, both PXRD patterns, simulated from single-crystal data, were mostly similar in the number of peaks, position, and relative intensities (Figure 3). This indicates that the presence or absence of the metal in the pyrrole ring hardly causes any changes in the lattice or the crystallographic structure.

### 3.3 Atomic and Molecular Composition

**Figure 4:**
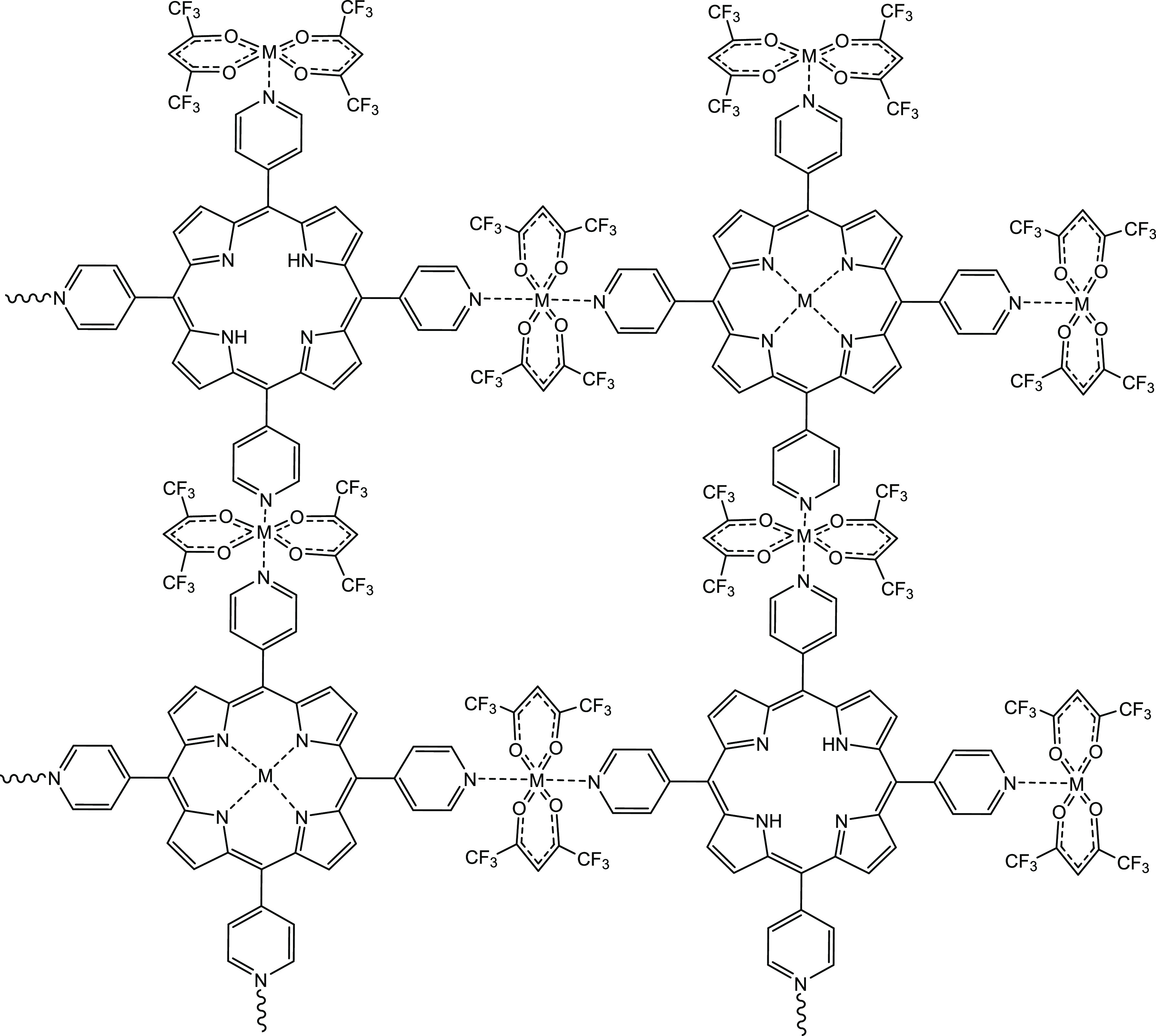
Scheme of the generic coordination of [M-TPyP]_*n*_ MOFs.

To further ascertain the presence or absence of the metal in the inner ring of the [M-TPyP]_*n*_ MOFs synthesized in scCO_2_, the composition was measured by performing elemental analysis for C, H, and N atoms and ICP for the metal. Analyzed data indicates the precipitation of materials with intermediate atomic proportions with respect to the values expected for fully free-base or metalated porphyrin (Table S2). Often, these results do not indicate the precipitation of a mixture of two crystallographic MOF phases, one with the pyrrole coordinated and one noncoordinated, but rather correspond to a single compound with the free-base form of porphyrin and the metalloporphyrin evenly mixed along the structure. Different results were obtained depending on the metal complex used for sample preparation. Considering that all the derived porphyrins underwent exocyclic coordination with M(hfac)_2_ at the four pyridine moieties, the coordination of the metal inside of the ring could only be partial for Cu(II), Zn(II), and Co(II) MOFs, as it is schematized in Figure 4. For Cu(II) and Zn(II), ca. 30% of the pyrrole rings were coordinated by a metal, while for Co(II), the metalloporphyrin form reached 70% likely due to the high solubility of Co(hfac)_2_ in scCO_2_ that favors the metalation of the porphyrin. In another situation, for Ni(II), an excess of metal of ca. 3.5 wt % was measured in the derived porphyrin MOF, even considering full exocyclic and endocyclic coordination. Taking into account the low solubility of Ni(hfac)_2_ in scCO_2_, the measured metal excess was attributed to the presence of the end product of the unreacted reagent.

**Figure 5:**
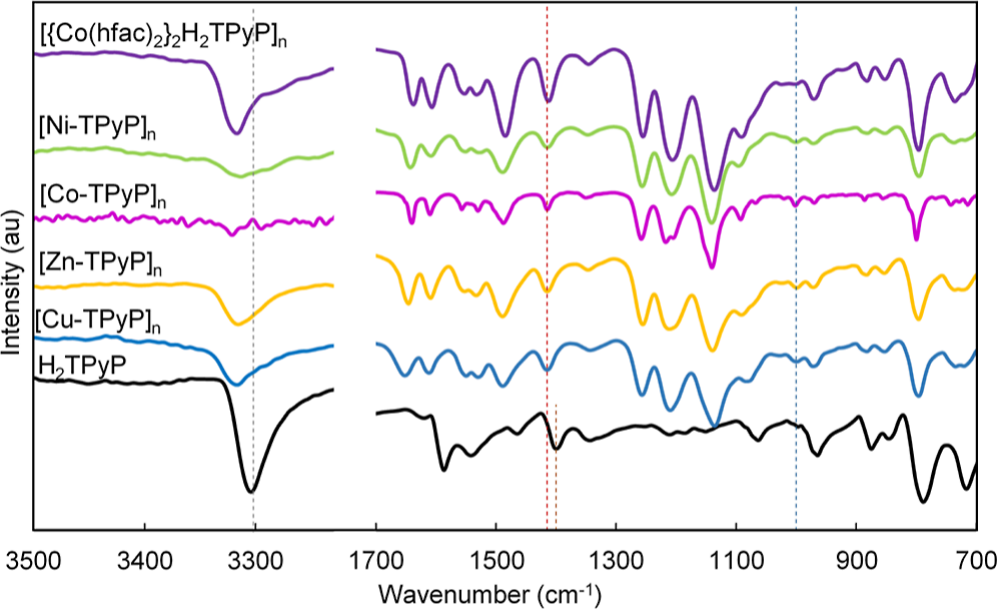
FTIR spectra of [M-TPyP]_*n*_ MOFs compared to pure H_2_TPyP.

The FTIR spectra of the synthesized [M-TPyP]_*n*_ MOFs are shown in Figure 5. All the scCO_2_ synthesized samples displayed similar bands. At high wavenumbers, the signal at 3300 cm^–1^ corresponds to the N–H stretching of the inner pyrrole ring. This band was highly reduced in intensity with respect to free H_2_TPyP and shifted to higher wavelengths upon complexation with the metal. Besides, a new band emerged corresponding to the N–M bond at ∼1000 cm^–1^, indicating that some metal is inserted into the porphyrin ring.

The bands at 1636 and 1200 cm^–1^ were assigned to C=O of the acetylacetonate in hfac and C=N, respectively. The bands at 1690–1540 and 1380 cm^–1^ were assigned to C=C and C–N stretching vibrations, respectively. The C=N band at 1400 cm^–1^ of the free pyridine was for the MOFs that slightly shifted to higher wavelengths (ca. 15 cm^–1^). The two bands at ∼1644 and 1600 cm^–1^ were assigned to ν(CO^–^) of the ligand, confirming that the metal complex ligand (hfac group) was not released upon assembly of the metal to the pyridine group. The crystalline and semiamorphous Co(II) MOFs present slight differences in the FTIR spectra. Essentially, the signal at 1000 cm^–1^, corresponding to the N(inner pyrrole ring)–Co bond in the semiamorphous [Co-TPyP]_*n*_, is absent in the sample obtained from layering with the formula [{Co(hfac)_2_}_2_H_2_TPyP]_*n*_.

### 3.4 Thermogravimetric Analyses

**Figure 6:**
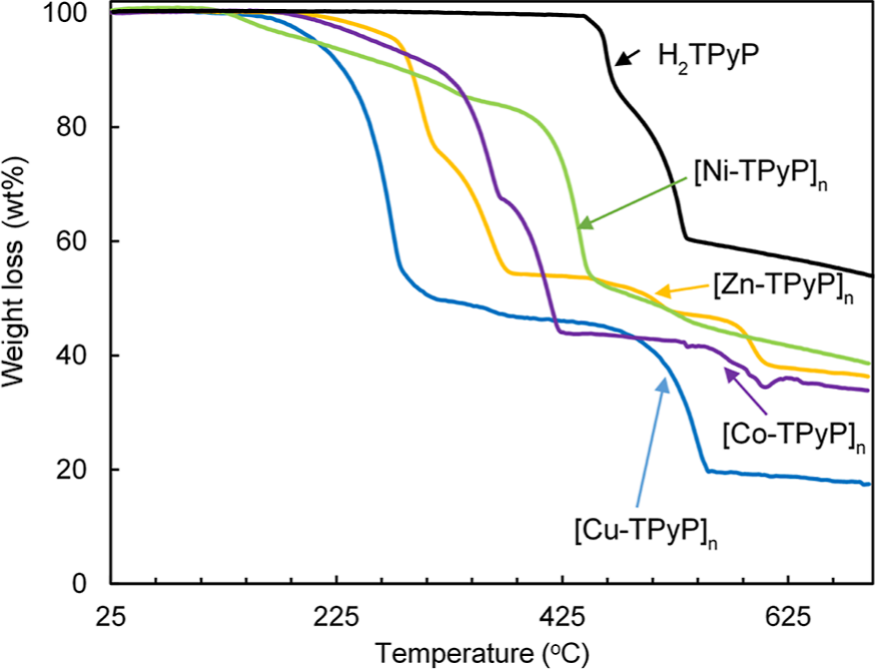
TGA curves of pure H_2_TPyP and [M-TPyP]_*n*_ MOFs.

The thermogravimetric decomposition curves of the different MOFs were compared to that of the free H_2_TPyP (Figure 6).

H_2_TPyP decomposition started at an onset temperature of 450 °C, ending at 700 °C, with a two-stage weight loss. The first (19 wt %, 450–475 °C) is attributed to the loss of the peripheral pyridine groups, and the second (25 wt %, 475–540 °C) corresponds to the decomposition of the tetrapyrrole ring. The ratio of these to weight loss values is close to the theoretical values of pyridine (50 wt %) and tetrapyrrole (50 wt %) molecules in the porphyrin. The total weight loss was only of ca. 50 wt % up to 800 °C. The residue has been attributed to the formation of thermally stable carbides. A comparison of the decomposition temperature of pristine H_2_TPyP with H_*x*_TPyP in the different MOFs reveals that the former has considerably more thermal stability than the corresponding metal counterparts. This behavior has been already described for metalated Zn(II), Cu(II), Ni(II), and Co(II) tetra(*p*-carboxylic acid phenyl)porphyrins.^6^ All MOFs showed initial weight losses in the range of 125–190 °C, attributed to the evaporation of the hfac. This was followed by several weight loss steps assigned to thermal decomposition, varying from metal to metal, which is attributed to the heterogeneity of the network structures. The final residue, varying from 38 wt % in Ni(II) to 25 wt % in Cu(II) MOFs, was assigned to the formation of metal oxides. From the studied compounds, the gradation of thermal stability was Ni(II) > Co(II) > Zn(II) > Cu(II) MOFs. This behavior has already been described for other porphyrin-related materials and is related to the size of the ionic radius of the metal.^37^

### 3.5 Morphological and Textural Properties

**Figure 7:**
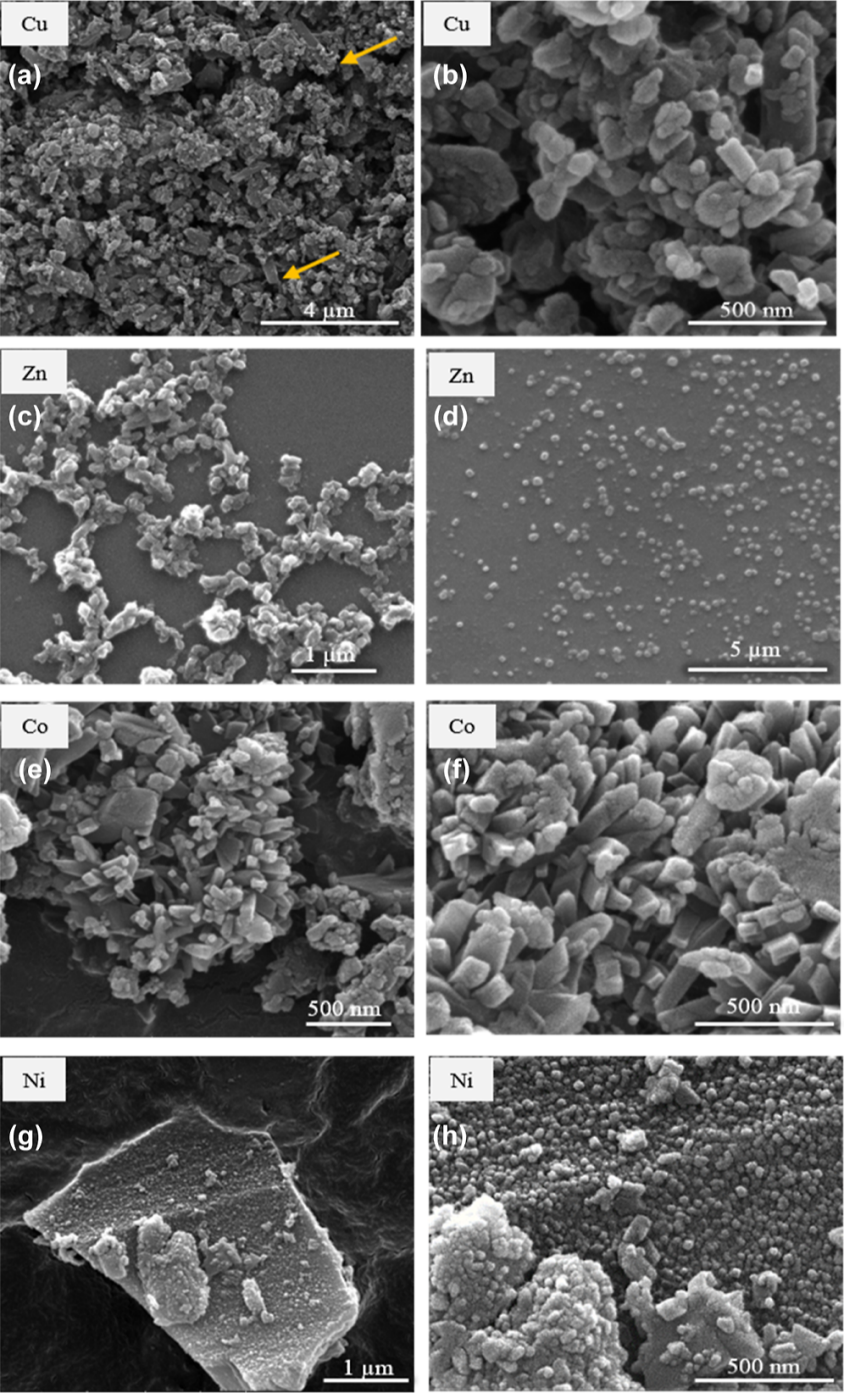
SEM images of [M-TPyP]_*n*_ MOFs: (a,b) Cu(II), (c,d) Zn(II), (e,f) Co(II), and (g,h) Ni(II).

The morphology of the precipitated MOFs was observed by SEM (Figure 7). For all the samples, the size of the precipitated particles was in the nano- to low-micrometer range. Cu(II) (Figure 7a,b) and Zn(II) (Figure 7c,d) MOFs precipitated as crystalline particles of heterogeneous diameters, from nano- to microsizes, where Cu(II) grew in an elongated way and Zn(II) in a very well-dispersed form. The hydrodynamic diameter was evaluated for the Zn(II) MOF by DLS, giving a mean value of ca. 140 nm (Figure S3). Crystals obtained from Co(II) (Figure 7e,f) precipitated with a large size as columnar crystals with defined edges.

This is attributed to the enhanced solubility of Co(hfac)_2_ in scCO_2_ when used together with the CHCl_3_ cosolvent, which also favored the solubility of the organic linker H_2_TPyP and, thus, the crystal growth. A completely different scenario was observed when using Ni(hfac)_2_ as the metal source. As it was assessed, Ni(hfac)_2_ is the least soluble in scCO_2_ of all the studied metal complexes, which appears to have an effect on the precipitation of the MOF. The Ni(II)-based MOF contains numerous nanometric entities (Figure 7g,h). In this case, nucleation is clearly favored over crystal growth. This fact is reflected in the precipitation of nanometric (<20 nm) particles, resulting in a poorly defined PXRD (Figure 3). To improve the crystallinity of this MOF, the use of CHCl_3_ as a cosolvent was attempted, but the final product was not suitable for characterization as a large amount of pyridine linker, unfeasible to be separated, remained unreacted. Only a few SEM images are reported in the literature showing the morphology of similar compounds obtained in organic solvents. Well-formed structures, namely, microlumps and microprims, have been described for the coordination of zinc-10,15,20-tetra(4-pyridyl)porphyrin with Cu(OAc)_2_ but need the use of cetyltrimethylammonium bromide for adjusting the coordination reaction and improving the crystal growth.^40^

Textural properties were obtained by measuring N_2_ adsorption–desorption isotherms at low temperatures (Table 1). Despite the different compositional and morphological characteristics measured for the scCO_2_-precipitated porphyrin-derived MOFs, all of the compounds had a similar N_2_ adsorption behavior, showing type I isotherms assigned to microporous products (Figure S5).

**Table 1:** Textural Properties of the Different Porphyrin MOFs Synthetized in scCO_2_

sample	*S*_BET_(m^2^ g^–1^)	*S*_Langmuir_(m^2^ g^–1^)	*V*_mp_(cm^3^ g^–1^)
[Cu-TPyP]_*n*_	460	490	0.31
[Co-TPyP]_*n*_	480	520	0.36
[Zn-TPyP]_*n*_	480	520	0.34
[Ni-TPyP]_*n*_	480	535	0.32

For the samples prepared using scCO_2_, theoretical surface areas could not be measured due to uncertainties in the structure definition and a large number of defects in the semiamorphous compounds. These samples were evaluated experimentally.

The BET surface area values were consistently derived at ∼480 m^2^ g^–1^, with a micropore volume of ∼0.30–0.35 cm^3^ g^–1^. This is a clear indication that, independently of the ratio of the metalized ring, the textural properties were not compromised by this fact. On the contrary, the textural properties of the Co(II) MOF obtained from layering could not be measured due to the low amount of sample obtained using this technique.

In this case, the theoretical surface area was calculated from the representation of the crystal structure, taking into account the void size. The pore shape was adjusted to a cylinder, using an equivalent diameter of 9.1 Å. This led to a theoretical estimated surface area of ∼1700 m^2^ g^–1^. Using this value, the calculated cell volume corresponds to 814 Å^3^, equal to the crystallographic value. Compared to published data for other H_2_TPyP MOFs, the only value of surface area found reported was for the compound involving the paddlewheel Cu(II) tetraacetate [Cu_2_(AcO)_4_], which was in the order of 800 m^2^ g^–1^.^22^

### 3.6 Photodynamic Characterization

[Zn-TPyP]_*n*_ was tested in this work as a potential PDT compound because Zn(II) has been reported to be highly biocompatible in metalloporphyrins.^38^

#### 3.6.1 UV–Vis Spectroscopy

**Figure 8:**
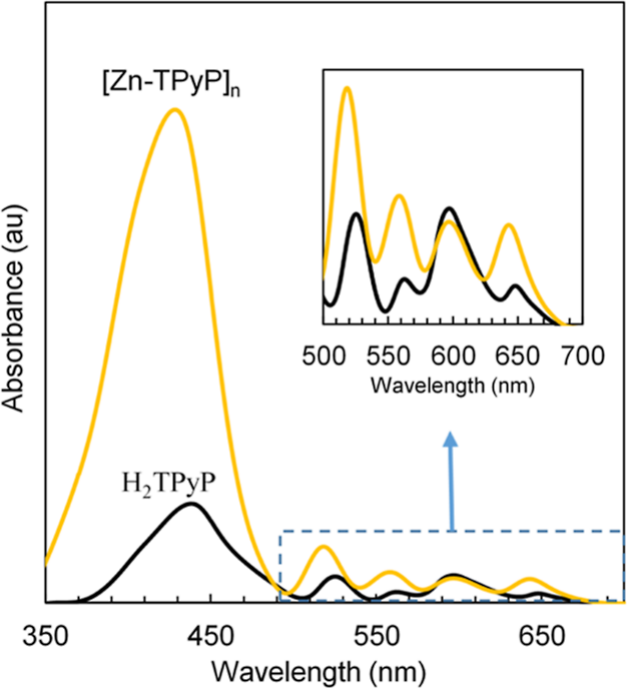
UV–vis absorption spectra of H_2_TPyP and [Zn-TPyP]_*n*_.

UV–vis spectroscopy is often used to understand porphyrins’ behavior. However, due to the potential lability of the N-metal bond, preliminary stability studies must be carried out. For this, a sample of the Zn(II) porphyrin MOF was dispersed in water by ultrasonication. The filtered and dried powder was analyzed by PXRD (Figure S4). Comparison with untreated samples indicates that the treated powder remained intact, demonstrating that this MOF was sufficiently stable to undergo water treatment, at least under the used experimental conditions. Red light with a wavelength of 630 nm is generally used for PDT since it corresponds to the region of the wavelength used in low-level laser therapy.^39^ Hence, the first step to analyze the suitability of the material for PDT studies is to perform UV–vis characterization. The intensity and color of porphyrins are derived from the highly conjugated π-electrons, giving characteristic UV–vis spectra that consist of two distinct regions in the near ultraviolet and in the visible region. Changes in the conjugation of a particular porphyrin would affect the UV–vis absorption spectrum. In this study, the spectra of [Zn-TPyP]_*n*_ and net H_2_TPyP were compared (Figure 8). The organic linker (black line) presents the typical porphyrin broad intense Soret band at λ_max_ = 437 nm. This band is present at a relatively high value of absorbance since the molecule is mesosubstituted, thus corresponding to the transition from the ground state (S0) to the second excited state (S2). The second set of less intense bands are the typical four weak Q bands in the range of 500–700 nm with phyllo-type intensities, corresponding to the transition S0 → S1 of the free-base porphyrin.^41^ Upon complexation with Zn(II) (yellow line), the Soret band shifted considerably to blue with a λ_max_ = 428 nm, which is attributed to the loss of symmetry in the porphyrin ring due to deprotonation of the central pyrrole nitrogen atoms and further metalation. In addition, the large complexation of the outer pyridyl groups with the metal can also provoke this large shift.^42^ It has been described that upon full complexation of the porphyrin with the metal in the central ring, the UV–vis spectrum also changed with respect to the Q bands since the four bands in H_2_TPyP corresponding to the NH vibrational excitations get reduced to two due to the increase in the symmetry in M-TPyP. The spectra of the Zn(II) porphyrin MOF synthesized in scCO_2_ displayed an intermediate situation, in which the four Q bands remained in the spectrum but with modified intensities, as it is observed in Figure 8. This result further indicates that, throughout the [Zn-TPyP]_*n*_ MOF network, the pyrrole ring was only partially metalized. The fact that the porphyrin MOF preserves the Q bands, and especially the band at 640 nm, is of extreme importance for its study to use the material as a potential PDT sensitizer.

#### 3.6.2 Measurement of Singlet Oxygen (^1^O_2_) Production

To demonstrate that the [Zn-TPyP]_*n*_ MOF has the potential to be tested in PDT, an assessment of singlet oxygen production was carried out following two different approaches. First, the fluorescence decay caused by the photobleaching of anthracene-9,10-dipropionic acid in the presence of singlet oxygen was measured. Second, quantification was performed by determining singlet oxygen quantum yields, based on the phosphorescence of ^1^O_2_ at 1270 nm.

**Figure 9:**
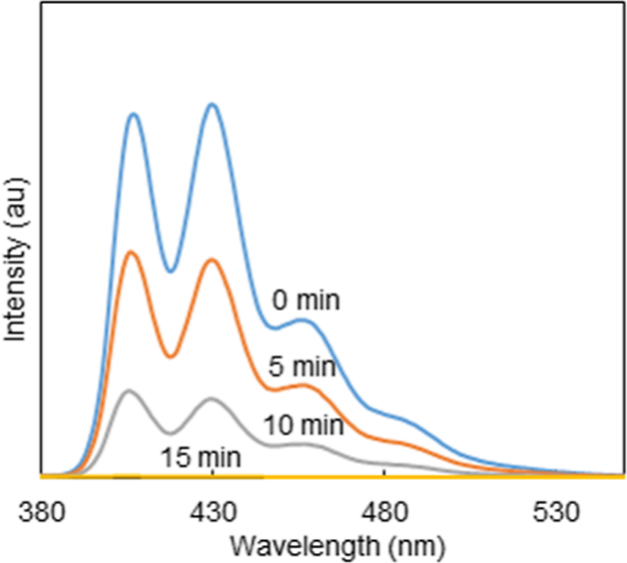
Fluorescence emission spectra of ADPA after irradiating for 15 min at 630 nm in the presence of the [Zn-TPyP]_*n*_ photosensitizer.

The fluorescence decay of the ADPA upon irradiation was monitored in time to detect the formation of singlet oxygen (Figure 9). The same light source, operating in the same conditions (630 nm, 55 mW cm^–2^ intensity) as in the PDT measurements, was used for irradiation. The wavelength of the irradiation closely corresponds to the typical Q-band of the porphyrin, nearest to the red region. An important decrease in the fluorescence intensity was detected already after 5 min, showing the photobleaching of the ADPA to the nonfluorescent endoperoxide^43^ in the presence of the irradiated photosensitizer. After 15 min, no fluorescence signal was detected, and the ADPA was irreversibly quenched. This result confirms the potential of [Zn-TPyP]_*n*_ MOF to be used as a photosensitizer in PDT treatment.

In a second set of experiments, the generation of singlet oxygen was performed by the direct measurement of its well-known phosphorescence at 1270 nm. Perinaphtenone was used as the standard reference in order to quantify this process. As expected, similar values for the dispersion of pure porphyrin crystals (ϕ_Δ_ = 2.7%) and the Zn(II) MOF (ϕ_Δ_ = 2.3%) was detected, although in the first case, the large H_2_TPyP crystals do not disperse homogeneously in the solvent, unlike the case of the MOF. These values were found to be quite low, which is the result expected in aqueous dispersions where, in comparison with other solvents, the concentration of the dissolved O_2_ is low.^44,45^ Nevertheless, aqueous media were used in order to maintain similar conditions to the PDT experiment. Despite the relatively low quantum yields, based on the important fluorescence decay, and the well-known photosensitizing ability of porphyrins, this experiment demonstrates that the [Zn-TPyP]_*n*_ MOF is a compound with characteristics adequate to be used in PDT.

### 3.7 Photodynamic Treatments

#### 3.7.1 Product Toxicity in Dark Conditions

**Figure 10:**
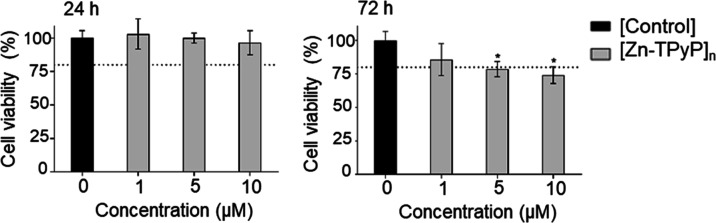
Dark toxicity: cell viability of SKBR-3 cells incubated for 24 h in dark conditions with different concentrations of the product (1, 5, and 10 μM) and a control (0 mM) at 24 and 72 h. * indicate statistically significant differences in cell viability compared to the control at each time point. Experiments were done in triplicate.

Once it was proven by UV–vis that [Zn-TPyP]_*n*_ can be used in PDT, the toxic effects of the product on cell viability, in the absence of light irradiation (dark toxicity), were evaluated using the Alamar Blue assay at two different time points as the amount of fluorescence produced is proportional to the number of living cells. As can be seen in Figure 10, none of the concentrations tested resulted in cytotoxicity after 24 h postincubation, but after 72 h of incubation, cell viability was significantly lower with 5 (78.6%) and 10 μM (74.1%) concentrations. Toxicity was concentration-dependent; the higher the concentration, the lower the viability.

#### 3.7.2 Cell Viability after Photodynamic Treatments

**Figure 11:**
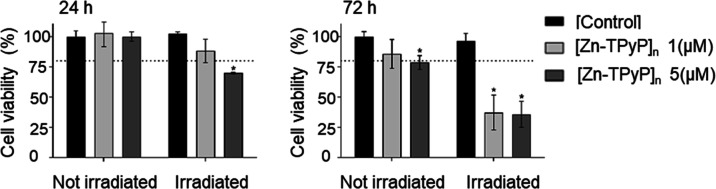
Photodynamic treatment effects after 15 min of irradiation. Cell viability was determined by Alamar Blue for SKBR-3 cells incubated without (control) or with 1 or 5 μM product for 4 h followed by cell wash. Cell viability was determined after incubation (24 h) either in dark conditions (not irradiated) or after 15 min of irradiation at λ_ex_ 620–630 nm (irradiated) and at 72 h. Three independent experiments were performed for each set of conditions. * indicate statistically significant differences in the cell viability between the control and product at each time point and condition. Experiments were done in triplicate.

Photodynamic treatments were performed with 1 and 5 μM concentrations of products because higher concentrations resulted in toxicity at 72 h postincubation in dark conditions. Photodynamic treatments were executed in an irradiator dispositive that homogeneously irradiates all the cells. Irradiation was performed at room temperature, which can affect cell viability. For this reason, dark toxicity was also tested under these conditions. To confirm that phototoxicity was due to product internalization, the product was incubated for only 4 h, followed by an exhaustive wash to eliminate all the noninternalized product (Figure 11); then, cells were irradiated for 15 min.

The results obtained in dark toxicity under these conditions were similar to that obtained in the first experiment; that is, the product was not toxic at any time point tested, except for 5 μM product at 72 h after product incubation (78.6%). After 15 min of irradiation, when incubating with 5 μM product, cells showed a significant decrease in cell survival after both 24 and 72 h (69.9 and 35.7% viability, respectively). On the other hand, incubation with 1 μM product only led to a significant decrease in cell survival after 72 h (37.2% viability). These results are in agreement with that obtained previously using the same SKBR-3 cell line and a porphyrin linked to Zn(II) metal complex (Na-ZnTCPP);^46^ for example, using 1 μM of this product, cell viability was reduced to more than a half. Other authors have also reported a reduction in cell viability using water-soluble porphyrins linked to the Zn(II) metal complex.^47^

### 3.8 Internalization of the Photosensitizers

**Figure 12:**
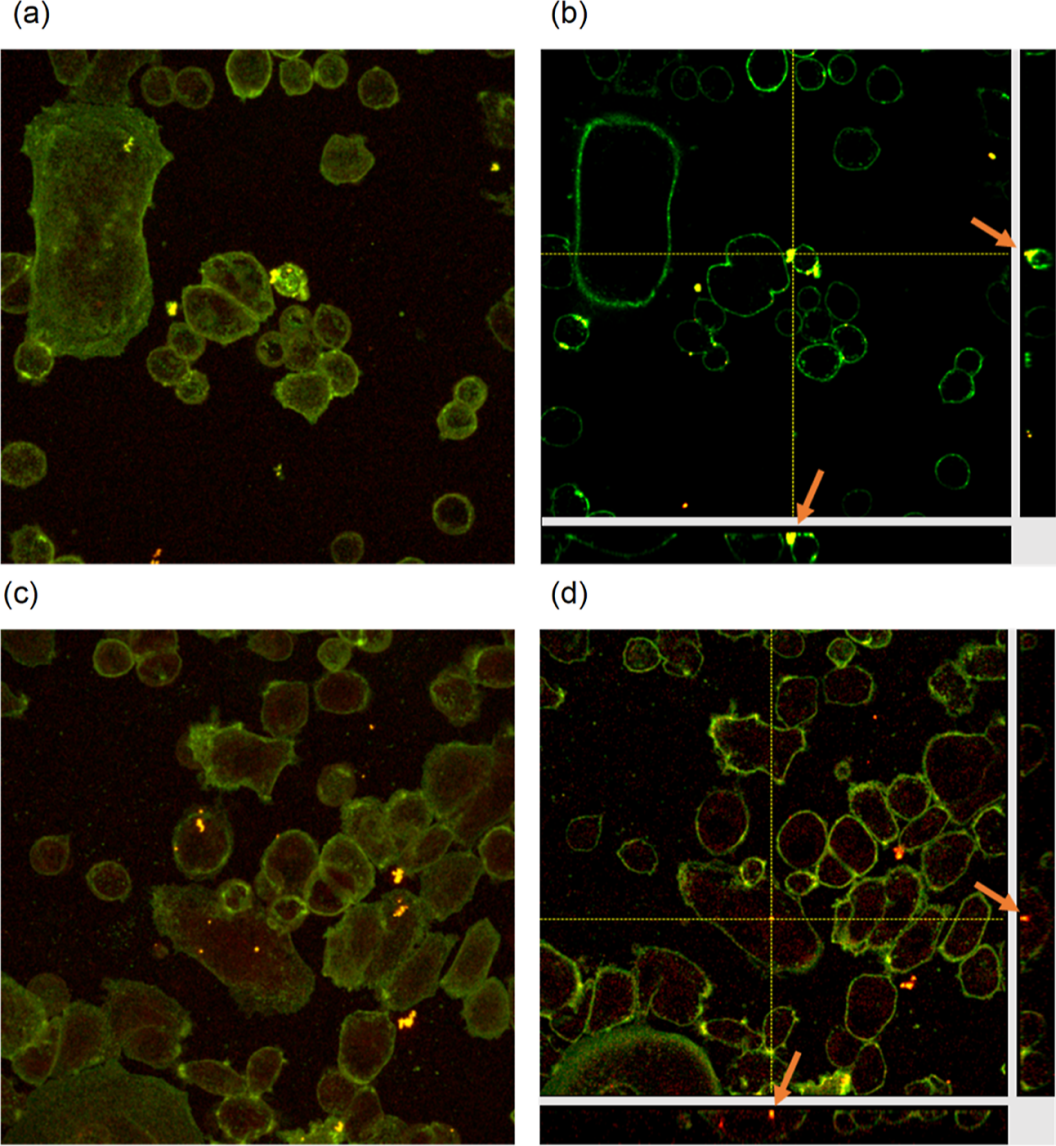
Live SKBR-3 cells incubated with 1 μM product for 4 h (a,b) and 24 h (c,d) and observed under a confocal microscope. To analyze the localization of the product, fluorescence mode was used. Product fluorescence emission was detected in the range of λ 600–763 nm (orange) by exciting the cells using a λ 405 nm laser (15% of the laser power). WGA fluorescence emission (membrane) was detected in the range of λ 500–550 nm (green) by exciting the cells using a λ 488 nm laser (12 1% of the laser power). Maximum projection (a,c) and orthogonal projection of z-stacks (b,d). Arrows point to some aggregates where the cross is positioned (b,d). Scale bar 20 μm.

Internalization of the product was analyzed by confocal microscopy. SKBR-3 cells were incubated with 1 μM product for 4 and 24 h, washed four times to eliminate all the noninternalized products, and incubated with WGA-488 for 15 min to visualize the plasma membrane (limit of the cell). Consecutive optical slices throughout the cell volume were taken to obtain the 2D maximum projection (Figure 12a,c), whereas orthogonal sections allowed to locate the product within the limits of the cell (Figure 12b,d). After 4 h of incubation, the product could be detected as a red background inside every single cell in the maximum projection (Figure 12a), but only aggregates were visible in the orthogonal projection (Figure 12b, arrows). However, after 24 h of incubation, product internalization was clearly observed in both images, maximum (Figure 12c) and orthogonal (Figure 12d) projections. Nevertheless, 4 h of incubation was enough to kill more than 50% of the cell population after 15 min of irradiation.

Considering that [Zn-TPyP]_*n*_ has been shown to generate ^1^O_2_ and that confocal studies have shown that [Zn-TPyP]_*n*_ could be effectively internalized by cells, it is assumed that the reduced cell viability during light irradiation is due to the generation of ROS. [Zn-TPyP]_*n*_ exhibits high phototoxicity and low dark toxicity at a concentration of 1 μM. This photosensitizer is highly efficient in inducing cell death, more than 62% after 72 h of a tumorigenic human mammary epithelial cell line. Other authors have demonstrated the use of different photosensitizers for PDT as an anticancer therapy.^46−49^

## 4 Conclusions

scCO_2_ proved to be a rapid, green, and effective approach for preparing MOFs from highly insoluble organic ligands. In this way, a collection of [M-TPyP]_*n*_ [M = Cu(II), Zn(II), Co(II), and Ni(II)] MOFs was prepared using scCO_2_ as reaction media using mild conditions. Elemental analysis, PXRD patterns, FTIR, and UV–vis characterizations indicated that MOFs obtained from Cu(II), Zn(II), Ni(II), and Co(II) were obtained as porous networks, where the full coordination of all binding sites was not complete. In addition, [{Co(hfac)_2_}2H_2_TPyP]_*n*_ was obtained from layering, whose structure was elucidated from SCXRD. This MOF was characterized for having a porous network from which the inner pyrrole ring remained uncoordinated. From all of the MOFs obtained, that of Zn(II) was used for PDT. The uncompleted coordination of the inner pyrrole ring gave place to the presence of a Q-band at 640 nm, which was key for targeting the MOF as a potential photosensitizer.

## Supporting Information Available

Reaction products from the conventional synthesis of MOFs; UV–vis spectra of complexation experiments of pure porphyrin with Zn(II); crystal data of [{Co(hfac)_2_}H_2_TPyP]_*n*_; particle size of [Zn-TPyP]_*n*_; PXRD confirming the stability of [Zn-TPyP]_*n*_; BETs of scCO_2_ obtained MOFs; and formulas and elemental analyses of MOFs (PDF)

## Accession Codes

CCDC 2190259 contains the supplementary crystallographic data for this paper. These data can be obtained free of charge via www.ccdc.cam.ac.uk/data_request/cif, or by emailing data_request@ccdc.cam.ac.uk, or by contacting The Cambridge Crystallographic Data Centre, 12 Union Road, Cambridge CB2 1EZ, UK; fax:+44 1223 336033.
