# Supplementary material for: Supercritical CO2 Synthesis of Porous Metalloporphyrin Frameworks: Application in Photodynamic Therapy
Source: Chem Mater. 2023 Jan 30;35(3):1080–93. doi: 10.1021/acs.chemmater.2c03018 (PMC9933429; doi:10.1021/acs.chemmater.2c03018)
Supplement: Supplementary file 1 — cm2c03018_si_001.pdf [file cm2c03018_si_001.pdf]

Supporting information

Supercritical CO_2 synthesis of porous metalloporphyrin frameworks: application in photodynamic therapy

Márta Kubovics,[1] Oriol Careta,[2] Oriol Vallcorba,[3] Guillermo Romo-Islas,[4,5] Laura Rodríguez,[4,5] Jose A. Ayllón,[6] Concepción Domingo,[1] Carme Nogués*[2] and Ana M. López-Periago*[1]*

[1] Institute of Materials Science of Barcelona (ICMAB-CSIC), Campus UAB s/n, 08193 Bellaterra, Spain. *amlopez@icmab.es, *conchi@icmab.es

[2] Department de Biologia Cel·lular, Fisiologia i Immunologia. Universtitat Autònoma de Barcelona (UAB), Campus UAB s/n, 08193 Bellaterra, Spain. *carme.nogues@uab.cat.

[3] ALBA Synchrotron Light Source, 08290 Cerdanyola del Vallés, Spain.

[4] Department of Inorganic and Organic Chemistry, Barcelona University, Martí i Franquès 1-11, 08028 Barcelona, Spain.

[5] Institute of Nanoscience and Nanotechnology (IN2UB), Barcelona University, Campus UB s/n, 08028 Barcelona, Spain.

[6] Department of Chemistry, Universitat Autònoma de Barcelona (UAB), 08193 Campus UAB s/n, Bellaterra, Spain.

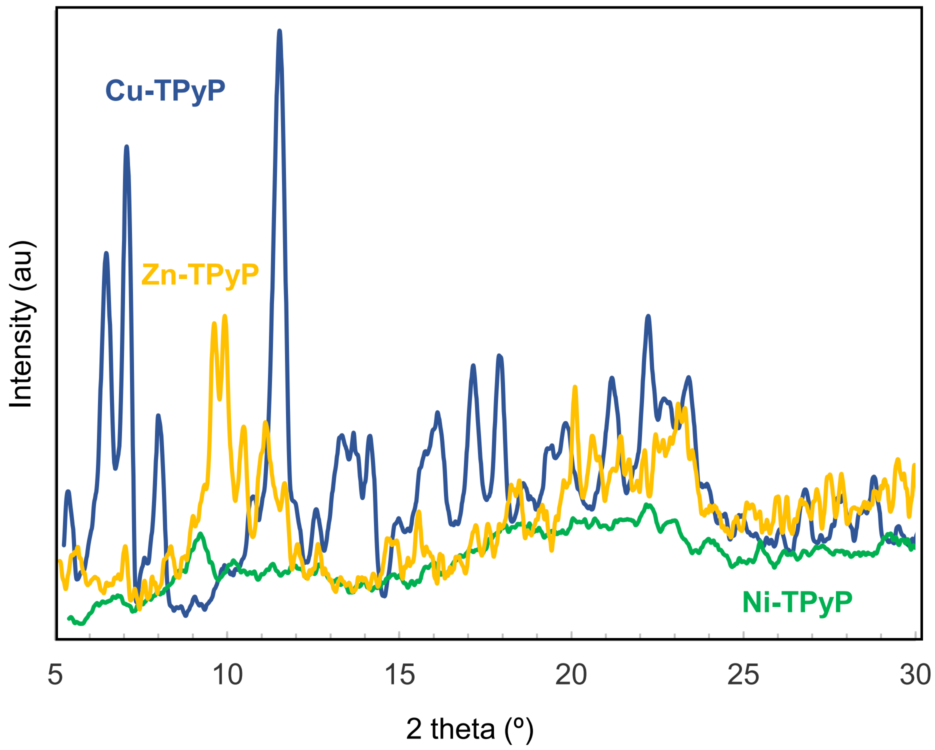

Figure S1. Capillary PXRD patterns of reaction products from the layering runs of Cu(II), Zn(II) an Ni (II) reacted with H_2TPyP.

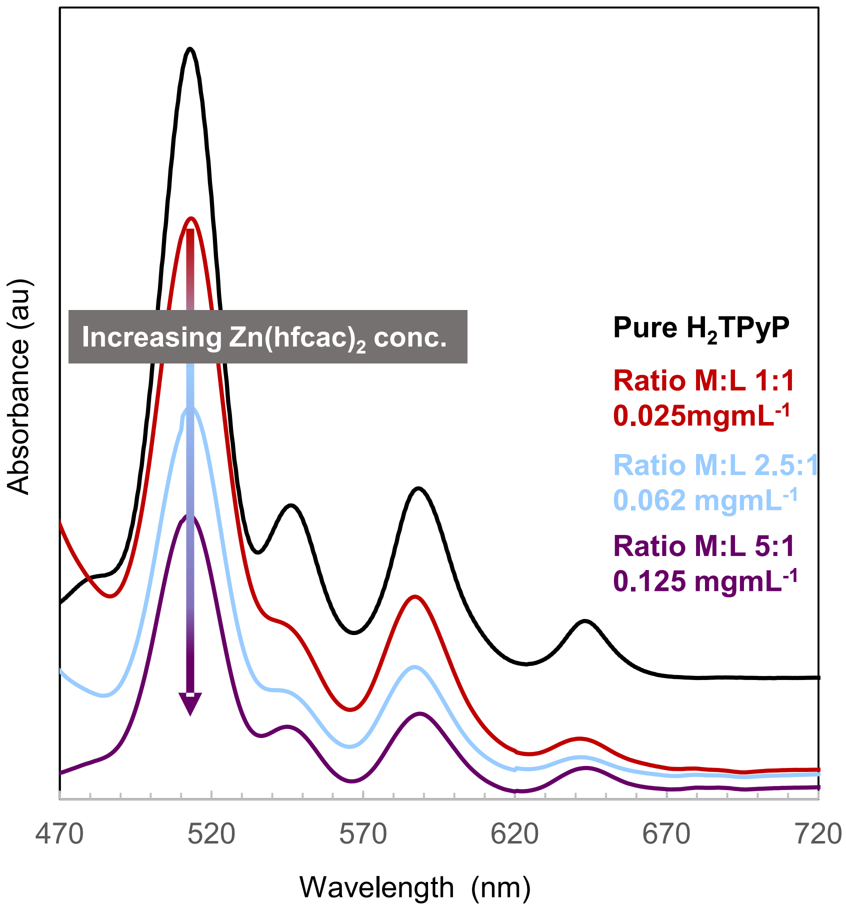

Figure S2. Titration experiments of H_2TPyP with $Zn(hfac)_2$. The porphyrin concentration was 0.025 $mgmL^{-1}$. The concentrations for the metal were 0.025 $mgmL^{-1}$ (red), 0.0625 $mgmL^{-1}$ (blue) and 0.125 $mgmL^{-1}$ (purple).

Table S1. Crystallographic data for compound $[\{Co(hfac)_2\}H_2TPyP]_n$ determined by single crystal diffraction.

**Compound**	$[\{\mathbf{Co(hfac)_2}\}\mathbf{H_2TPyP}]_\mathbf{n}$
Empirical formula	$C_{15}H_{7.5}CoF_6N_2O_2$
Formula weight	391.19
Radiation	X-rays
T (K)	100(2)
Wavelength (Å)	0.72932
System, space group	Monoclinic, *C 2/m (12)*
a (Å)	22.913(10)
b (Å)	25.721(4)
c (Å)	6.524(1)
β (°)	105.60(2)
V ($Å^3$)	3703.25
Z	8
*D*calc (g cm^{-3})	1.403
μ (mm^{-1})	0.597
F(000)	1560
Crystal size (mm^3)	0.100x0.060x0.040
hkl ranges	$-31 \leq h \leq 31$, $-35 \leq k \leq 35$, $-8 \leq l \leq 8$
2θ range (°)	1.248 to 29.967
Reflections collected/unique/[R_{int}]	125748/5021/[R_{int} = 0.0546]
Completeness	98.6%
Refinement method	Full matrix least-squares on $\|F\|^2$
Data/restrains/parameters	5021/0/234
Goodness of fit (GOF) on $\|F\|^2$	1.134
Final *R* indices [$I > 2\sigma(I)$]	R1 = 0.0483, wR2 = 0.1401
R indices (all data)	R1 = 0.0500, wR2 = 0.1416
Largest. Diff. peak and hole (e $Å^{-3}$)	0.945 and -0.995

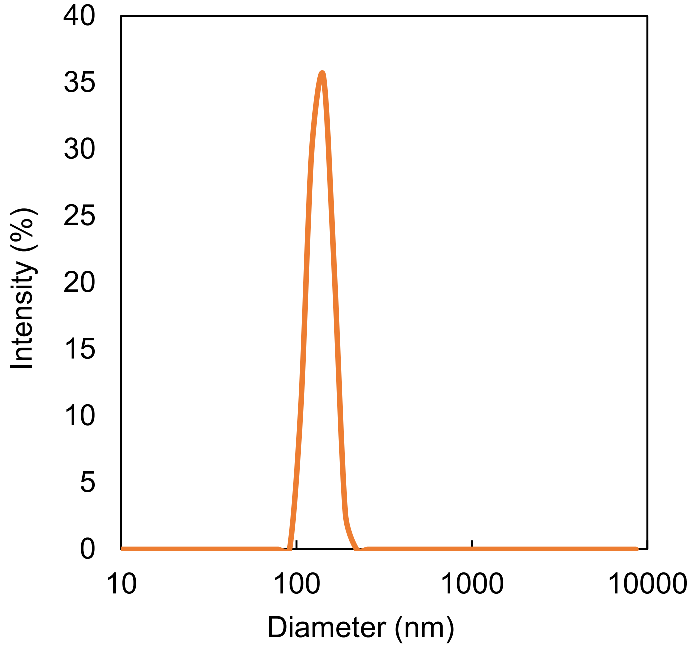

Figure S3. DLS measurement of $[Zn\text{-}TPyP]_n$ sample in water.

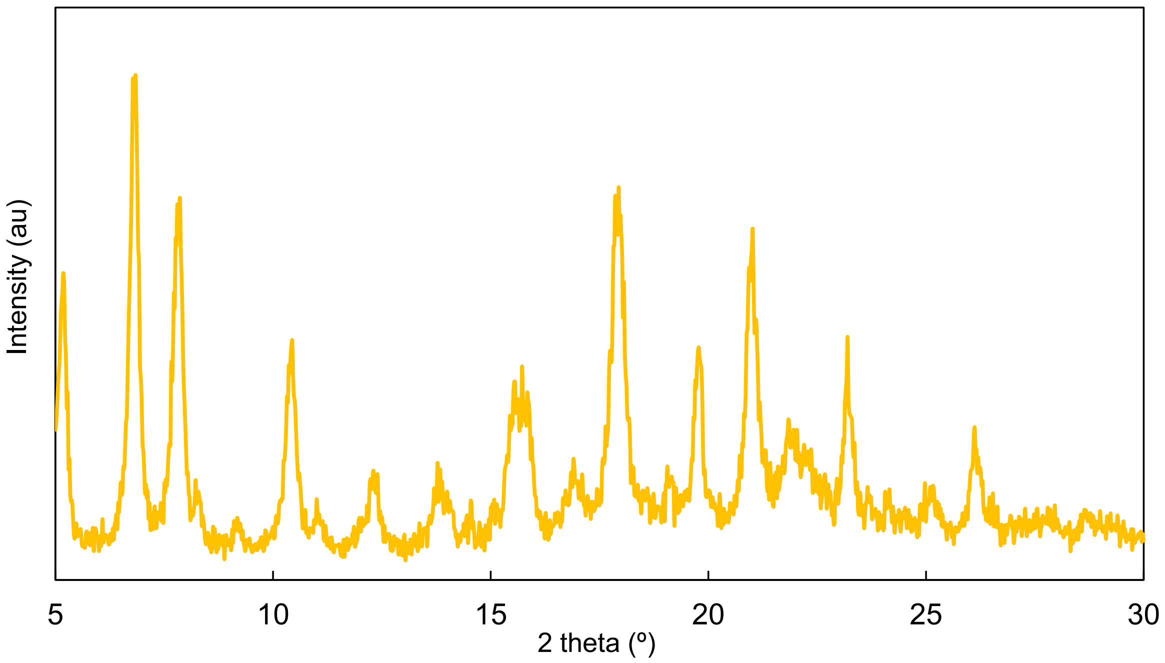

Figure S4. PXRD of the $[Zn\text{-}TPyP]_n$ sample after its dispersion in water achieved applying sonication.

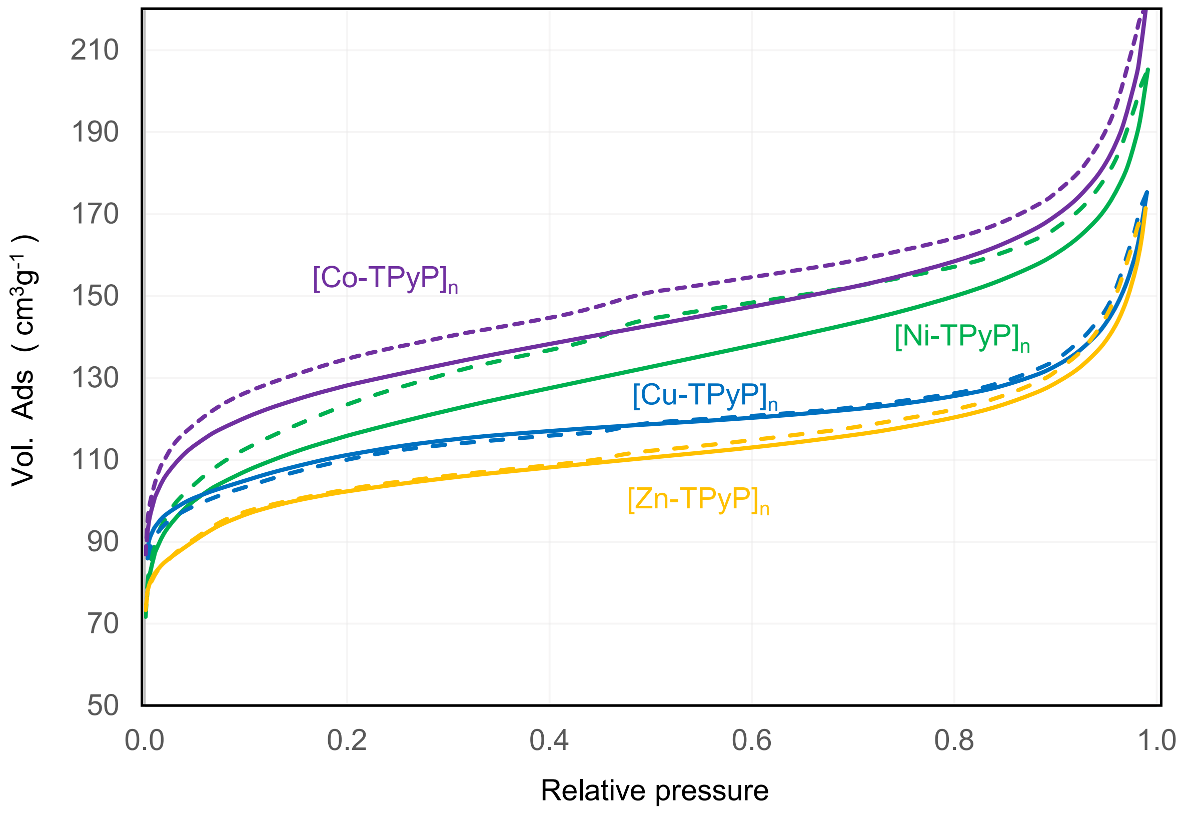

Figure S5. N_2 adsorption-desorption isotherms at -196 ºC for all samples.

Table S2. Experimentally measured C, H and N molar percentages obtained by elemental analysis, presented together with metal molar percentages by ICP for the samples precipitated in $scCO_2$.

Sample	Atom	Exp. data	Derived viable formula
$[Cu\text{-}TPyP]_n$	C	46.44	$[(Cu(hfac)_2)_2Cu_{0.32}\,(H_2)_{0.67}TPyP]_n$
	H	1.96	1.0 % H_2TpyP
	N	6.99	
	Cu	9.22	
$[Zn\text{-}TPyP]_n$	C	46.74	$[(Zn(hfac)_2)_2Zn_{0.30}(H_2)_{0.70}TPyP]_n$
	H	1.94	
	N	7.40	
	Zn	9.31	
$[Co\text{-}TPyP]_n$	C	44.45	$[(Co(hfac)_2)_2Co_{0.70}(H_2)_{0.30}TPyP]_n$
	H	2.00	
	N	6.40	
	Co	9.24	
$[Ni\text{-}TPyP]_n$	C	44.64	$[(Ni(hfac)_2)_2NiTPyP]_n$
	H	1.67	3.5 % $Ni(hfac)_2$
	N	6.63	
	Ni	14.31	
